# Domoic acid affects brain morphology and causes behavioral alterations in two fish species

**DOI:** 10.1038/s41598-023-49041-0

**Published:** 2023-12-08

**Authors:** Kassandra Beltrán-Solís, Ernesto García-Mendoza, Samuel Sánchez-Serrano, Lus M. López

**Affiliations:** 1 Posgrado en Ecología Marina, Centro de Investigación Científica y de Educación Superior de Ensenada, Baja California (CICESE), Ensenada, Mexico; 2 Departamento de Oceanografía Biológica, Centro de Investigación Científica y de Educación Superior de Ensenada, Baja California (CICESE), Ensenada, Mexico; 3 Facultad de Ciencias Marinas, Universidad Autónoma de Baja California (UABC), Baja California, Mexico

**Keywords:** Behavioural ecology, Ichthyology

## Abstract

Domoic acid (DA) produces neurotoxic damage in seabirds and marine mammals when they are exposed to this potent neurotoxin. Other vertebrates are also susceptible to DA intoxication including humans. However, neurobehavioral affectations have not been detected in fish when naturally exposed to DA but only when it is administered intraperitoneally. Therefore, the current idea is that fish are less sensitive to DA acquired under ecologically relevant routes of exposure. Here, we show that oral consumption of DA induces neurobehavioral and histopathological alterations in the brain and heart of totoaba (*Totoaba macdonaldi*) and striped bass (*Morone saxatilis*). Lesions were found in both species in the optic tectum and cerebellum after exposure for 7 days to a diet containing 0.776 µgDA g^−1^. The affectations prevailed chronically. Also, we found that cardiac tissue exhibits lesions and focal atrium melanism. Although affectations of the brain and heart tissue were evident, excitotoxic signs like those described for other vertebrates were not observed. However, the use of standardized behavioral tests (dark/light and antipredator avoidance tests) permitted the detection of behavioral impairment of fish after DA exposure. Pathological and associated behavioral alterations produced by DA can have relevant physiological consequences but also important ecological implications.

## Introduction

Domoic acid (DA) is a toxin produced by marine algae, particularly diatoms of the genus *Pseudo-nitzschia*. When the toxin is present in the environment, filter-feeding organisms can accumulate DA and transfer it to upper trophic levels^1,2^. Accumulation of DA in different organisms represents a risk to animals and can cause the Amnesic Shellfish Poisoning (ASP) in humans. DA is a chemical analog of glutamate that binds with high affinity to receptors of this neurotransmitter when it crosses the blood–brain barrier (BBB)^3,4^ causing overactivation of the receptors that leads to neuronal death and neurotoxic signs such as confusion, disorientation, scratching, seizures, coma, and even death in birds and mammals^3,5^. Besides excitotoxic behaviors induced by DA, this toxin generates histopathological alterations in the central nervous system of different groups of vertebrates^6^. DA intoxications have been reported in humans^7^, birds^8–10^, and piscivorous marine mammals^9,11,12^ but not in fish. Fish are vectors of DA to seabirds and marine mammals since they can accumulate high levels of the toxin during toxic *Pseudo-nitzschia* blooms^13^. However, there is no evidence that fish are affected by DA under natural conditions of exposure. Fish show neurotoxic affectations when DA is administered intraperitoneally (IP) but not when the toxin is incorporated via oral uptake, that is the ecologically relevant route of exposure^13^. Salmon (*Oncorhynchus kisutch*)^14^, rainbow trout (*Oncorhynchus mykiss*)^2,14^, and anchovy (*Engraulis mordax*)^5^ exposed orally to DA did not show excitotoxic behavior even when the toxin was accumulated in the brain, liver, and kidney. Therefore, fish are believed to be less sensitive to orally acquired DA even though they have similar susceptibility at the receptor level as in marine mammals and birds^14^. Here, we reevaluate this idea. We investigated the effect of DA on fish after a mortality event of adult *Totoaba macdonaldi* breeders that were housed in the Wildlife Management and Conservation Unit for *Totoaba macdonaldi* of the Autonomous University of Baja California (UABC). Analysis of fish food revealed the presence of DA, evidencing chronic exposure of the fish to the toxin. Therefore, to understand any potential impacts of this toxin on the health of the fish, we performed an oral gavage bioassay with the species. Additionally, DA-contaminated diet bioassays were performed with totoaba and with striped bass (*Morone saxatilis*) juveniles. Behavior was documented, and DA neurotoxic effects on these species were assessed through behavioral tests and histopathological analysis.

## Results

### Effect of DA injected intraperitoneally

Four dosing levels (0.8, 1.6, 3.2 and 6.4 µgDA g^−1^) were each administered intraperitoneally (IP) to triplicate *T. macdonaldi* groups to evaluate the neurological susceptibility of the organisms to the toxin. All organisms exposed to ≥ 1.6 µgDA g^−1^ presented disorientation and swam in circles, spirals, or upside down (See Supplementary Video 1). These signs were registered after 35.3 ± 5.1 min for the 1.6 µgDA g^−1^ and 30.3 ± 8.5 min for the 3.2 µgDA g^−1^ dose. None of the injected individuals exposed to these DA doses died during an observation period of 6 h. In contrast, similar neurotoxic signs were observed in organisms exposed to 6.4 µgDA g^−1^ at 16.3 ± 11.6 min and died at 49.3 ± 22.8 min after injection. The effect of the IP injection of DA was represented by clear and extensive damage in the brain and heart tissue. Organisms exposed to ≥ 1.6 µgDA g^−1^ presented large areas of neurons separated from the adjacent tissue, neuropil loss, and oncotic necrosis in cells in the optic tectum (OT) (See Supplementary Fig. 1). Furthermore, oncotic necrosis of Purkinje cells soma was detected in the cerebellum. Besides brain lesions, the heart tissue presented signs of cardiomyopathy represented by separation between muscle fibers in the myocardium and inflammatory cell infiltration (See Supplementary Fig. 2).

### Effect of DA administered orally

The micropippete-guided drug administration (MDA) method^15^ was used to evaluate the effect of the oral administration of DA on totoaba juveniles. DA dissolved in water (1.6 µgDA mL^−1^) was administered to a triplicate *T. macdonaldi* group. The fish did not exhibit neurotoxic behavior, indicating the potential likelihood of the toxin not reaching the brain. However, 0.084 ± 0.006 µgDA g^−1^ was quantified in brain samples two hours after administration. Toxin concentration diminished to 0.064 ± 0.001 µgDA g^−1^ 24 h after administration and at 72 h almost the same concentration was detected (Table 1). In contrast, 72 h after being administered, DA concentration diminished by 65% and 10% of the concentration detected at two hours in the liver and kidney, respectively (Table 1). Whereas in control organisms the toxin was not detected in the tissues. Like the tissular damage detected after the IP injection, the oral administration of DA caused damage to the brain and cardiac tissue. Necrotic neurons in the OT and neuropil loss in the brain tissue were observed. Also, separation between muscle fibers, necrotic cardiomyocytes, and inflammatory cell infiltration in the myocardium was observed in heart tissue (See Supplementary Fig. 3). The dose applied did not produce behavioral alterations like abnormal swimming patterns.

**Table 1:** Domoic acid (DA) tissue distribution in *Totoaba macdonaldi* after micropipette-guided drug administration of the toxin. The data is reported as mean ± standard deviation (n = 4).

	Time after exposure		
	2 h	24 h	72 h
Brain (µgDA g^−1^)	0.084 ± 0.006	0.064 ± 0.001	0.063 ± 0.01
Liver (µgDA g^−1^)	0.105 ± 0.007	0.07 ± 0.002	0.069 ± 0.005
Kidney (µgDA g^−1^)	0.719 ± 0.01	0.086 ± 0.004	0.072 ± 0.07
Total (%)*	56.75	13.75	12.75

*Of the total dose. If 100% of the toxin was absorbed via the oral gavage.

### Effect of DA incorporated by food consumption

**Figure 1:**
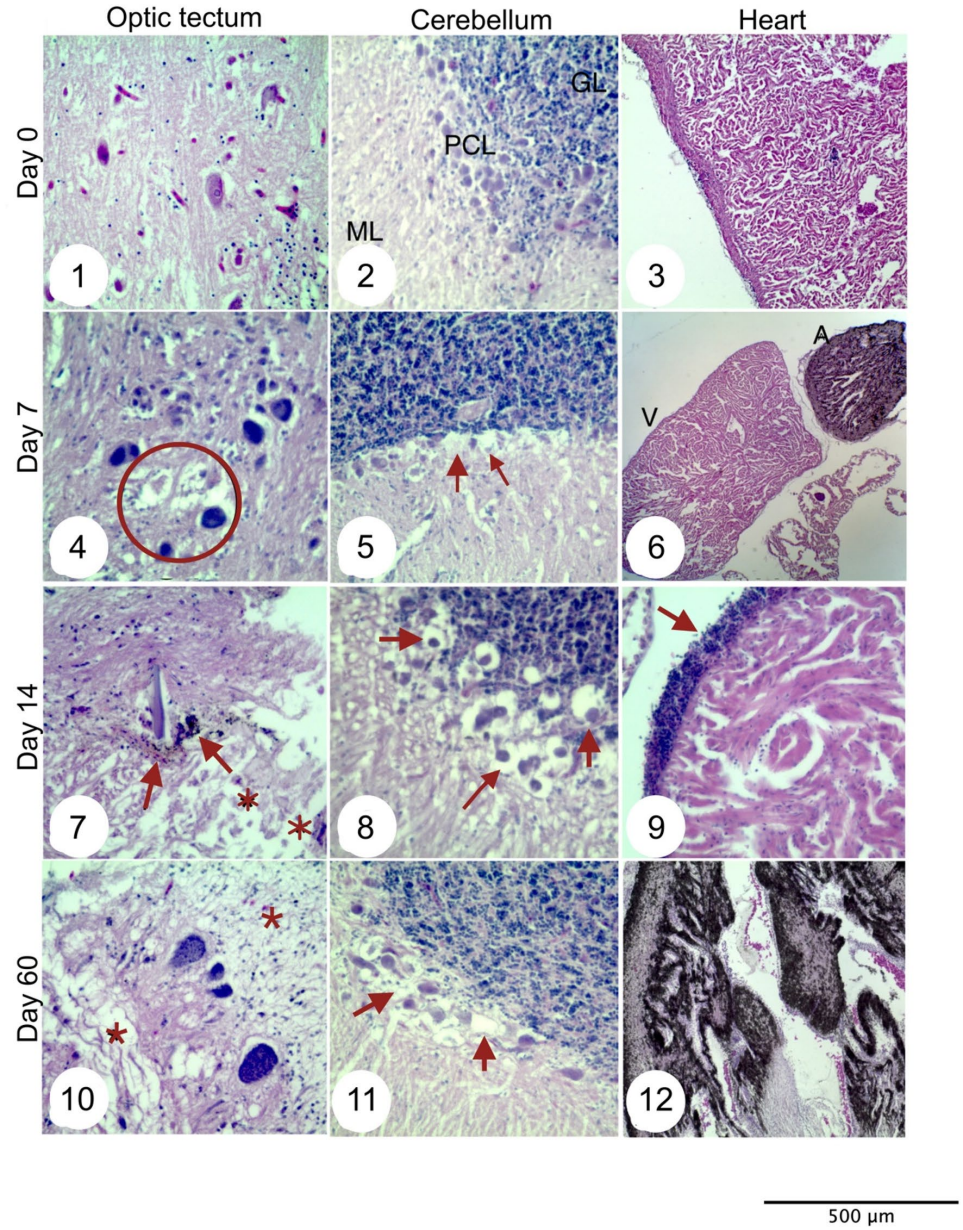
Brain and heart tissue alterations on *Totoaba macdonaldi* after oral consumption of DA. Optic tectum (OT) (1), cerebellum (2), and heart (3) without tissular damage at day 0. OT (4) showed neuropil loss and neurons with oncotic necrosis (circle), vacuolization at the Purkinje cell layer was evident in the cerebellum (5), and heart (6) presented atrial melanism (A) and ventricle (V) without alterations after 7 days of DA consumption. On day 14, the OT presented melanin granules outside the neurons (arrows) and neuropil loss (7), the cerebellum showed vacuolization of Purkinje cells (8), and nuclei accumulation at ventricle periphery and separation between muscle fibers was evident in the heart (9). Neurons with cytoplasmic granules (center of the image) and neuropil loss were present on day 60 in the OT (10). Vacuolization at Purkinje cells was still evident on this day in the cerebellum (11), and the heart showed atrium with melanism (12).

The effect of DA incorporated by a natural route of exposure was further evaluated on *T. macdonaldi* juveniles. The organisms were fed with a mixed mussel-fish slurry contaminated with DA at a low concentration (LC-diet: 0.114 µgDA g^−1^) and high concentration (HC-diet: 0.776 µgDA g^−1^). Also, striped bass (*M. saxatilis*) juveniles were exposed to DA to evaluate the susceptibility of another fish species to this toxin. No signs of affectation such as abnormal swimming or differences in the pattern of food consumption were registered in both species during the first days of exposure of the organisms to the two diets. The only noticeable effect was that striped bass stopped the consumption of the HC-diet (see Supplementary Video 2) 7 days after the beginning of the experiment and totoaba after 45 days. The organisms were fed with commercial pellets (EWOS salmon diet, Astra-Ewos, AB, Sweden) after the rejection the HC-diet and until the end of the experiment. The effect of DA on growth of the organisms was evaluated while they were consuming the HC-diet. No significative differences (t-student, p > 0.5) were found in weight gain, specific growth rate, and average daily weight gain (Table 2) between groups of organisms exposed to the two diets. The consumption of feed contaminated with DA also induced tissular lesions to the heart and the brain and induced changes in brain morphology (See next section). Organisms fed with the high DA content diet presented clear tissular lesions in the brain and heart. Lesions started in the optic tectum with oncotic necrosis and spongiform change of the neuropil after 7 days of exposure (Fig. 1). Alterations in the cerebellum were registered 14 days after the beginning of the trial. The tissue damage in the brain in both species continued to spread even after the cessation of consumption of the HC-diet. There was no recovery of the organisms even with cessation of consumption of contaminated feed. The change from contaminated feed to commercial pellets do not influence the results of the experiment. Besides brain lesions, both species exposed to the HC-diet showed focal cardiac melanism in the atrium (Fig. 16,12) with no other alterations detected in the heart tissue. Organisms fed with the LC-diet showed no tissular damage on the brain or heart tissue (See Supplementary Fig. 4).

**Table 2:** Growth parameters for striped bass and totoaba juveniles feed with two diets contaminated with domoic acid.

	Striped bass		Totoaba	
	LC-diet	HC-diet	LC-diet	HC-diet
Initial weight (g)	16.2 ± 3.93	16.91 ± 3.83	3.52 ± 0.87	3.43 ± 0.94
Final weight (g)	15.85 ± 3.71	17.71 ± 3.57	10.20 ± 3.45	13.04 ± 4.08
Weight gain (g)	− 0.35	0.8	6.68	9.61
Specific growth rate (%)	− 0.3120	0.6603	2.36	2.97
Average daily weight gain (g)	− 0.05	0.1142	0.15	0.21

#### Morphological brain alterations induced by DA acquired by food consumption

**Figure 2:**
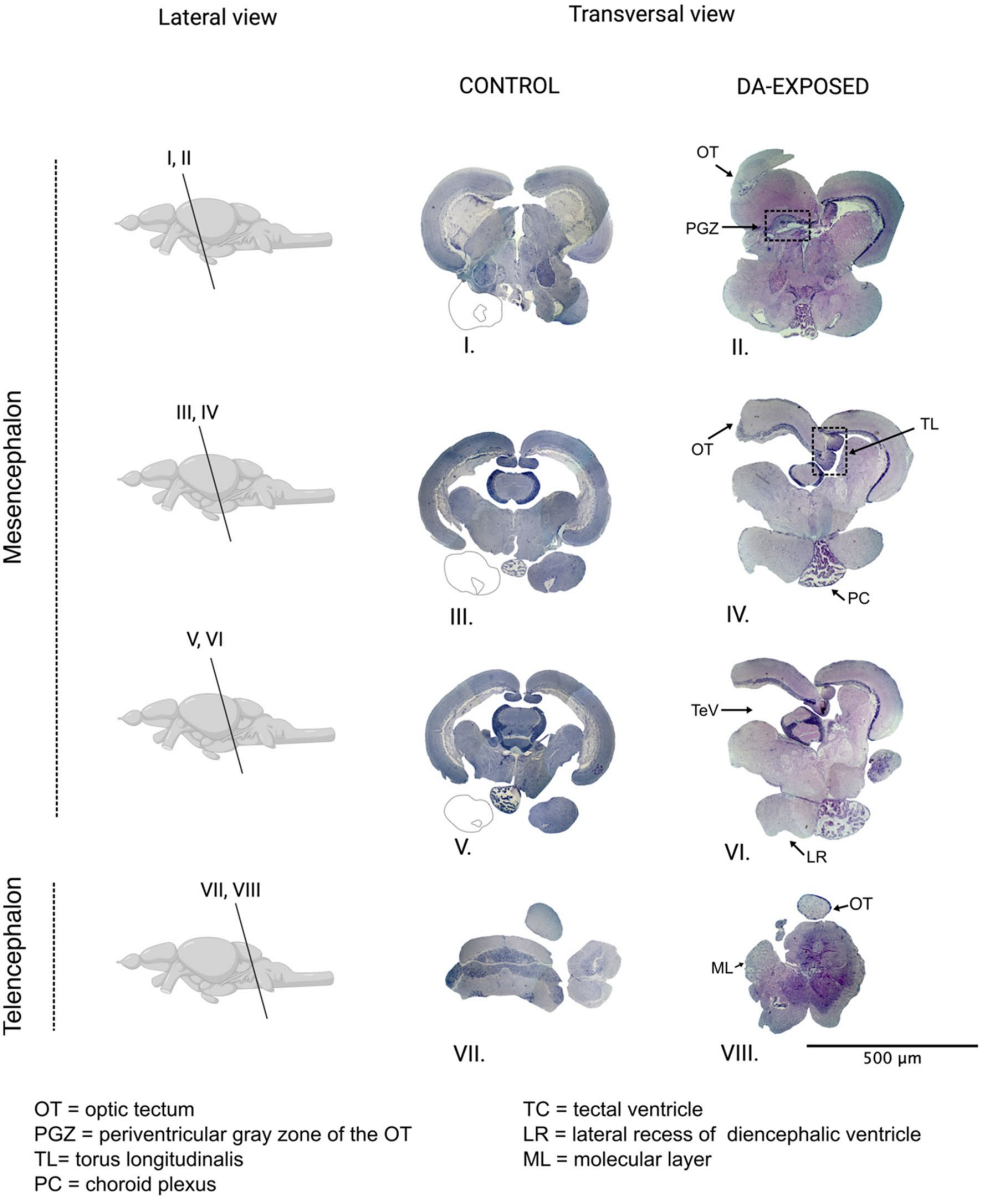
Comparison between serial sections of control and organisms fed with a DA-contaminated diet. The lateral view represents the general pattern of the brain in teleosts and the location of the cross sections presented on the right side of the figure (transversal view of the sections) is indicated with Roman numerals.

Related to the documented extensive tissular damage in the brain, the evaluation of its morphology was assessed to identify if DA could induce macroscale affectations to this organ. Serial cuts were generated on the mesencephalon and telencephalon because these were the regions where the most important tissular damage was identified in the histopathological analysis. Totoaba juveniles presented important alterations in brain morphology after being exposed to the HC-diet for 45 days. The most evident alteration observed was the loss of bilateral symmetry of the brain. Morphological damage induced by DA consumption in the totoaba was characterized by malformations in the right hemisphere (Fig. 2; Supplementary Figs. 7 and 8). As described above, most of the tissue damage was localized in the optic tectum (OT), and this region presented important macroscale structural changes. The OT folded externally and shows a reduction in the periventricular gray zone (Fig. 2II; Supplementary Fig. 8) at the most cephalic region of the mesencephalon (Fig. 2I,VI). The central regions of the telencephalon, presented a reduction of the OT surface (compared with non-affected brains) (Fig. 2IV,VI). Changes in the OT morphology were accompanied by the loss of integrity of the *torus longitudinalis* and *torus semicircularis* (in the most cephalic region) and a displacement of these structures towards the inner part of the opposite hemisphere (in the central region). Other evident morphological alterations induced by DA on the mesencephalon were the enlargement of the choroid plexus accompanied by the presence of blood cells (Fig. 2IV,VI) and an opening of the tectal ventricle and rupture of the lateral recess of diencephalic ventricle (Fig. 2VI). In the telencephalic region (Fig. 2VII,VIII) an alteration in the morphology of the *corpus cerebelli* was observed (Fig. 2VIII; Supplementary Fig. 8). This area of the brain of non-affected organisms showed an elongated shape (Fig. 2VII) whereas in the DA exposed organisms the *corpus cerebelli* presented a circular shape, as well as a displacement of the most caudal part of the OT to the upper part of the organ and an external folding in the molecular layer of the cerebellum (Fig. 2.VIII).

#### Behavioral tests

As mentioned, excitotoxic behavior induced by DA was not detected in the organisms exposed orally to this toxin. However, after the observation that DA induced important alterations in different brain regions, we assessed the possible neurobehavioral affectations on totoaba and striped bass juveniles exposed to DA through a learning test and standardized behavioral tests used to evaluate the effect of toxicants on fish.

##### Learning behavioral test

**Figure 3:**
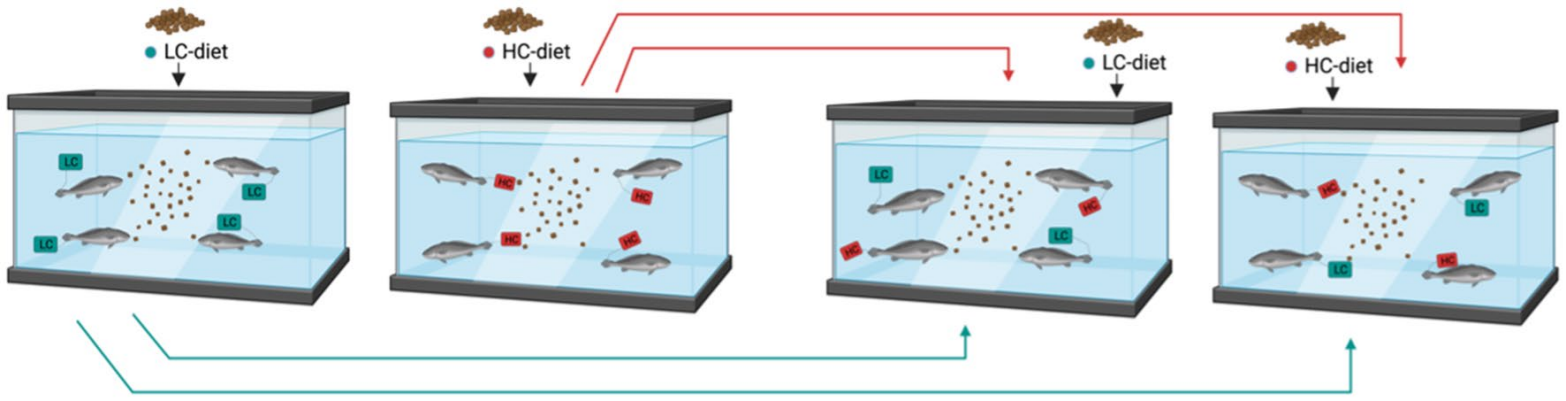
Learning behavioral test. Response of the organisms after being exposed to DA-contaminated fed. Organisms exposed to the HC-diet (red tag) were transferred to the aquarium with fish exposed to LC-diet. Organisms exposed to the LC-diet (blue tag) were transferred to the aquarium with the HC-diet. HC-fish started to consume the LC-diet after two days of exposure and continue to reject the high DA content diet. In contrast, LC-fish consumed both diets but rejected the HC-diet on day 7.

After the juvenile striped bass that received the HC-diet stopped its consumption we implemented a behavioral test to assess whether the cessation of food consumption was related to the detection of the presence of DA in the diet. Striped bass organisms that received the HC-diet and stopped the consumption (HC-fish) were exposed to the LC-diet whereas fish exposed to this last diet (LC-fish) were fed with the high DA content diet (Fig. 3). HC-fish started to consume the LC-diet after two days of exposure and continue to reject the high DA content diet. In contrast, LC-fish consumed both diets but rejected the HC-diet on day 7 (See Supplementary Video 3).

##### Dark/light box test

**Figure 4:**
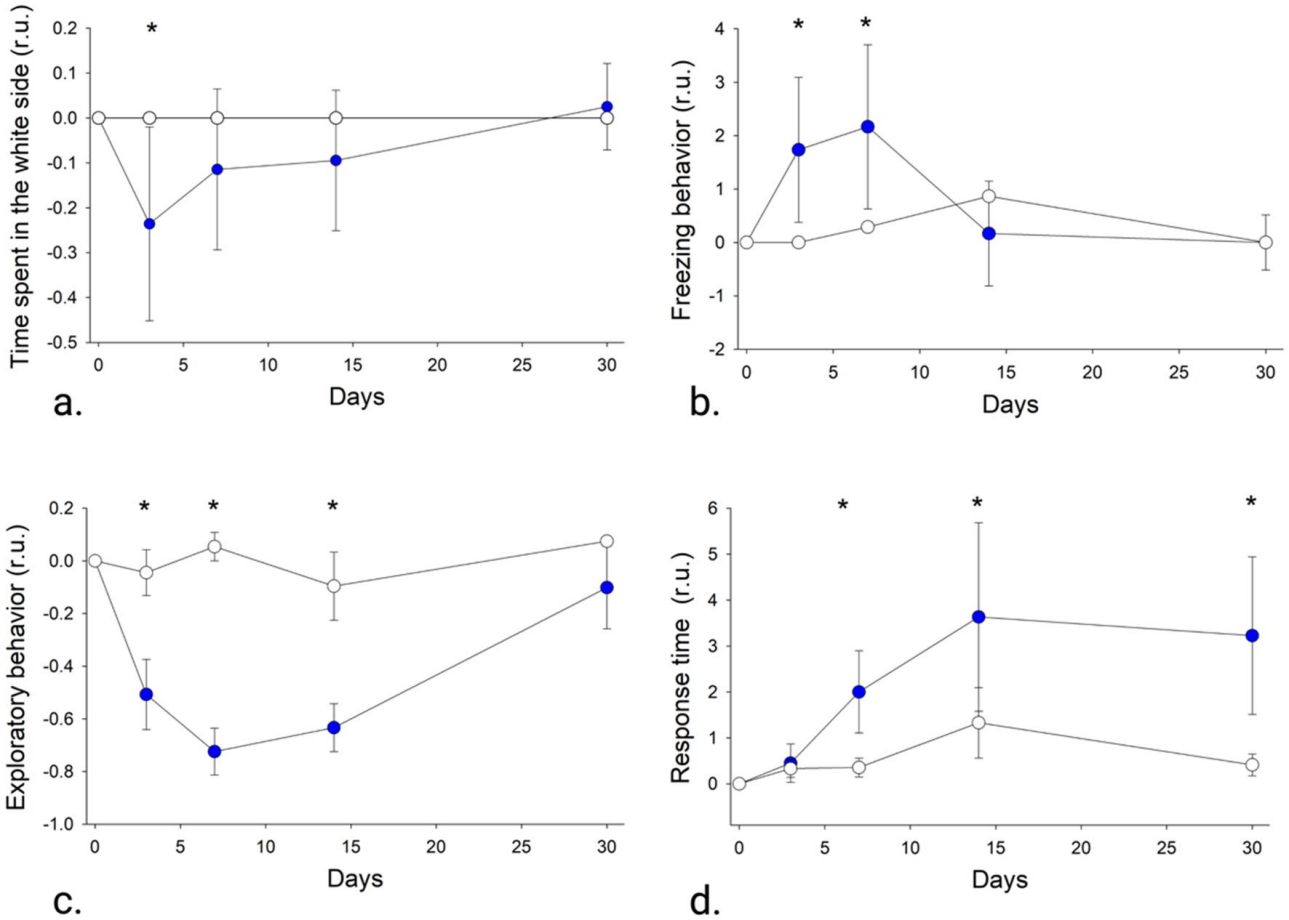
Behavioral biomarkers of *Totoaba macdonaldi* exposed (blue symbols) and unexposed (empty symbols) to domoic acid (DA). (**a**) Time spent on the white side of the trial aquarium. (**b**) number of freezing events. (**c**) Exploratory time during the trial. (**d**) response time to a hypothetical predator. The change (in relative units; r.u.) of each behavioral biomarker was assessed through the comparison of the response of the organisms at each sampling point to the one measured at day 0 before the beginning of the exposition to DA. The mean and standard error (n = 7) is presented. *Represents a significant difference in the behavioral biomarker of DA-exposed organisms at each sample point compared to the response to non-exposed organisms.

The dark/light box test is used to study anxiety on fish and it is based on the preference of some fish species for dark environments. This test also evaluates different behavioral biomarkers like exploratory behavior and freezing events in which slight alterations could be indicators of neurological damage. Organisms fed with commercial pellets showed a marked preference for the white side of the trial aquarium, spending all the time of the experimental session on this side (Fig. 4a). DA-exposed fish seemed to spend less time on the white side of the aquarium as compared to organisms fed with commercial pellets. This was evident 3 and 7 days after the exposure to DA (Fig. 4a). Also, on these days a significant increase in the number of freezing events and an abrupt decrease in the exploration time during the trial period was evident in DA-exposed organisms (t-student, p < 0.5; Fig. 4b). Exploration time on day 7 was approximately 80% lower than the one measured at the beginning of the experiment (day 0; Fig. 4c).

##### Antipredator behavior

Since two of the most important integrative centers for motor coordination (optic tectum and cerebellum) in the fish nervous system were affected by DA, escape time in response to a hypothetical predator was evaluated in DA-affected organisms. Fish exposed to DA had a slower escape response to the simulated predator attack than unexposed fish (t-student, p > 0.5; Fig. 4d). Thirty days after exposure to DA, the response time of affected tototaba juveniles was almost three times slower compared to non-affected organisms.

## Discussion

Here, we clearly demonstrated that DA acquired orally affects brain and heart tissue and induces behavioral alterations in two carnivorous fish species. The effect was not restricted to tissular damage, evident brain morphological changes were also detected in DA-exposed organisms. We documented similar tissue damage and the time of appearance of the lesions in the brain and heart of totoaba and striped bass. Chronic damage generated by DA acquired orally might be similar for other fish species, however the question still remains as to whether DA can significantly impact carnivorous and planktivorous fish species during blooms. Moreover, the use of standardized behavioral tests permitted the detection of subtle but important behavioral alterations caused by DA.

Although it has been established that fish are not affected by DA administered orally it is known that they are neurologically susceptible to this toxin. IP administration of DA causes alterations in the swimming behavior of salmon (*Oncorhynchus kisutch*), rainbow trout (*Oncorchynchus mykiss*), and gilt-head seabream (*Sparus aurata*)^14,16,17^, and even caused the death of anchovy exposed to ≥ 4.3 µgDA g^−1^^5^. We confirmed that totoaba and stripped sea bass juveniles (data not shown) presented alterations in their swimming behavior and died after being exposed to doses of 6.4 µgDA g^−1^. These behavioral alterations indicate that DA crosses the blood–brain barrier. DA reaching the brain was confirmed after the toxin was detected in this organ in anchovy, salmon, and gilt-head seabream after IP administration^5,14,17^, and after oral exposure in anchovy^5^. We also found the presence of DA in the brain of totoaba juveniles after oral exposure. DA concentration detected after 72 h of the oral administration indicates that the toxin that reached the brain can accumulate and remain in this organ at relatively stable levels over time whilst the toxin is rapidly eliminated from the kidney and liver.

Since DA reaches and could remain in the brain of fish it is expected that causes affectations to specific regions of this organ. However, alterations in brain tissue have not been described in any fish species after exposure to DA. Here we documented important lesions in the brain (and cardiac tissue) of totoaba exposed to DA via three different routes and in striped bass exposed to DA-contaminated feed. The effect was like that described in other groups of animals but not reported lesions were also identified in fish brains. The type of neuronal necrosis identified in totoaba and striped bass brains, was similar to that described in humans^6^, California sea lions^18^, southern sea otters^19^, northern fur seals^20^, and Pacific harbor seals^21^. The hippocampus is the area of the brain where DA induces the most significant damage in mammals^22,23^. This region has the highest density of glutamate receptors to which DA binds with high affinity^3^. In fish, this region of the brain has not evolved, and we found that in totoaba and striped bass, the primary target of DA was the optic tectum (OT). This suggests that the OT is the region with the major density of glutamate receptors in the fish brain. Another difference with the documented DA-associated tissular damage in mammals is that we detected clear vacuolization at the Purkinje cell layer of the cerebellum in the totoaba and striped bass brain. Alterations in the cerebellum have not been described for other organisms after toxin exposure to DA.

Besides the detection of focalized tissular lesions in the brain, we found important morphological changes of this organ in totoaba juveniles that consumed DA contaminated fed. DA caused a loss of symmetry of the brain with structural alterations focalized in the right hemisphere of this organ. Also, other areas of the brain with important functions, such as the *corpus cerebelli* and choroid plexus, showed significant structural alterations. The *corpus cerebelli* is one of the three parts of the cerebellum and is the entry point for cerebellar tract that carries cerebellar afferents from many stem nuclei^24^. Consequently, it is expected that alterations in this region might cause damage to the motor coordination of the organisms, as observed in totoaba (see below). Moreover, the choroid plexus is a structure that projects into the ventricles of the brain, secretes cerebral spinal fluid, and maintains the integrity of the BBB^24^. A change in the integrity of the BBB can make organisms more susceptible to toxic substances in the environment as they may enter the nervous system without the protection provided by this barrier. The corpus cerebelli and choroid plexus are structures shared by many vertebrate groups but there is no evidence that DA affects these specific regions in any animal model.

Like the brain, the heart of totoaba juveniles exposed to DA presented lesions described before in other organisms since glutamate receptors can be found in this organ. Specifically, necrotic cardiomyocytes and separation between muscle fibers described in affected California sea lions^18^ and southern sea otters^19^ were evident in totoaba and striped bass juveniles exposed to DA. However, we also found focal melanism in the atrium of DA-exposed organisms. Melanism is characterized by the presence of melano-macrophages, which are cells recruited to sites with chronic inflammation in ectothermic vertebrates^25–27^ which suggests an inflammatory process induced by DA consumption in fish.

Besides the description for the first time of tissular damage in the brain and heart associated with the consumption of DA-contaminated fed, we also documented important behavioral alterations in two fish species induced by the oral consumption of this toxin. Juveniles of totoaba and striped bass rejected the food contaminated with DA which was associated with a learned behavior as organisms were able to distinguish between diets with different DA content. The avoidance of phycotoxin-contaminated food seems not to be a rare behavior. The rejection of food contaminated with saxitoxins has been reported in sea otters^28^, and in the fish species *Salmo gairdneri* (rainbow trout) and *Salvelinus alpicus* (artic char)^29^. Similarly, organisms of the species *Seriola rivoliana* stop the consumption of okadaic acid-contaminated feed^30^. However, the detection or avoidance of DA-contaminated food by any organism has not been reported. The patterns of food rejection shown by the organisms indicate that this behavior is species-specific, and that *M. saxatilis* is more susceptible to DA.

Other behavioral alterations in totoaba juveniles were assessed through standardized tests to evaluate the neurological affectation of fish exposed to toxic substances. The light/dark test is used to study anxiety in fish since it is based on the natural preference for dark environments of some species^31–34^. In general, anxiolytic drugs increase the time spent by the organisms in the light compartment, while anxiogenic drugs decrease this time^35^. However, *T. macdonaldi* in culture prefers light environments. This was reflected in the experiment since control organisms and, to a lesser degree affected organisms, remained on the light side of the trial aquarium. Since totoaba is a carnivorous predator species, the bright side of the tank represents an environment where food might be found, and because the organisms used in the experiment were reared in culture facilities, they were probably not used to seek shelter. Anxiety in fish is characterized by freezing, hiding, an increase in movement, defecation, and thigmotaxis behavior^35^. The heavily affected exploratory behavior (increase in the number of freezing events and a decrease in time expended on this behavior) suggests that DA mimics the effect or acts as an anxiogenic drug. Moreover, the escape response in fish is an avoidance behavior mediated by neurons that receive vibrational stimuli and display muscle contraction as a response^35,36^. Since the OT and cerebellum as two of the most important integrative centers for motor coordination in the fish nervous system were affected, the escape response was consequently affected by the consumption of DA. Furthermore, several species of vertebrates show a general pattern of lateralization in several species, with the right hemisphere specialized in controlling social behavior, response to unexpected stimuli (e.g., predators), and processing global information^37,38^. The observed lesions in the right hemisphere of totoaba juveniles are probably related to the reduction in the scape response to a hypothetical predator considering that totoaba is a brain-lateralized species.

Although fish do not exhibit typical excitotoxic behavioral signs of DA poisoning such as those observed in marine mammals and birds, this study suggests there are underlying effects that may impact fish health. Here, we demonstrated that even in the absence of classic neurotoxic signs, totoaba and striped bass were affected by DA orally acquired. Evident histopathological alterations were found in brain and heart tissue, as well as subtle behavioral alterations were detected after exposure. Although there must be a species-specific behavior or susceptibility to DA, the fact that fish are susceptible to DA acquired through a natural route could have important ecological implications. For example, DA could make organisms more susceptible to predation in nature. Predator–prey interactions define the behavioral patterns of organisms and when a stressor disrupts these patterns there may be important ecological implications. DA can convert a natural predator organism into the prey of larger organisms. How DA can affect the ecological fitness of fish must be investigated.

## Methods

### Ethics declarations

1. All the procedures in this study were approved and were carried out in accordance with the care protocol from the Comité de Bioética para el Manejo de Organismos Vivos of the Ensenada Center Scientific Research and Higher Education (CICESE, Spanish acronym) (Código de Ética del Posgrado de CICESE, Art.II, item 7).
2. Our study followed the management ethical statutes that are mandatory in the activities of the Wildlife Management and Conservation Unit for *Totoaba macdonaldi* of the Autonomous University of Baja California (UABC) (DGVS-CR-IN-1084-B.C./09) and of Pacifico Aquaculture Co. (DGVS-CR-IN-1519-B.C./12) on the protection of cultivated organisms.
3. The optimal conditions were used for the conservation and good care of fish juveniles. All the methods are reported following the recommendations in the ARRIVE guidelines.

### Fish maintenance

Juvenile totoabas (*Totoaba macdonaldi*) (mean weight ± SD = 16.53 ± 3.79 g) and juvenile striped bass (*Morone saxatilis*) (mean weight ± SD = 13.47 ± 0.88 g) were used in the bioassays. The organisms were donated by The Wildlife Management and Conservation Unit for *Totoaba macdonaldi* of the Autonomous University of Baja California (UABC) and by Pacifico Aquaculture Co. of Baja California, Mexico. The organisms of each species were placed in 20-L aquariums equipped with cascade filters and aeration pumps and were maintained at 17 to 20 °C with a 14:10 (L:D) photoperiod. Water changes of 1/3 of the volume of each aquarium were performed daily. The organisms were acclimatized for 10 days before each trial (experiment). Fish were fed to satiety twice a day with commercial pellets (EWOS salmon diet, Astra-Ewos, AB, Sweden) during the acclimatization period.

For the behavioral tests, each individual organism represented an experimental unit. Therefore, each organism was identified with a mark in the caudal fin to be recognized in the experimental setups.

### Intraperitoneal injection

Four DA doses (0.8, 1.6, 3.2, and 6.4 µgDA g^−1^) were injected intraperitoneally to totoaba juveniles. Three fish were injected for each dose. Each organism was injected on the ventral side between the ventral fins and the anus using a 22-gauge needle. Also, 200 µL of sterile distilled water was injected to three organisms. After injection, fish were released into 20- L aquariums with natural seawater. The organisms were observed for 6 h.

### Micropippete-guided drug administration (MDA) bioassay

DA was administered using the MDA method to twelve totoabas (mean weight ± SD = 11.58 ± 2.28 g). A dose of 1.6 µgDA g^−1^ was administered to each organism to characterize the neurotoxic effects of the oral DA uptake. The DA was dissolved in 200 µL of sterile distilled water. Three additional organisms (mean weight ± SD = 12.34 ± 1.23 g) were gavage with 200 µL of distilled water only (control group). The 1.6 µgDA g^−1^ dose was selected because this amount of toxin induced neurotoxic signs without causing the death of the fish in the IP injection experiment (see results and discussion section). The organisms were liberated in a tank (one for control and one for DA-dosed fish) after the administration of DA. Organisms were sampled 2 h (n = 3), 24 h (n = 3) and 72 h (n = 3) after the administration of the toxin. One fish from the control tank was also sampled at the same time points. Organisms were euthanized and samples of different organs were collected for histopathological analysis and DA quantification.

### Diet bioassay

The effect of DA acquired orally present in an organic matrix was investigated by exposing totoaba and striped bass juveniles to two diets contaminated with the toxin. Organisms were fed with a mixed mussel-fish slurry contaminated with DA at a concentration of 0.114 µgDA g^−1^ (LC-diet) and 0.776 µgDA g^−1^ (HC-diet). Lyophilized mussel tissue contaminated with DA (CRM-ASP-Mus-D, National Research Council of Canada) was used to prepare the HC-diet. The CRM-Zero-Mus (National Research Council of Canada) was used to prepare the LC-diet. This material was mixed with ground sardine and powdered gelatin to obtain a solid paste. No true control was obtained since the sardines used in the mixed mussel-fish slurry contained low amounts of DA even in absence of bloom of DA-producing species. The exposure experiment was the comparation of two diets with different concentrations of the toxin. Each diet was hand-fed twice per day to organisms maintained in 20 L aquariums with natural seawater. The amount of feed was about 5% of the average weight of the organisms. The aquariums were cleaned daily, and fish were observed for unusual behavior. Two organisms were sampled at 7, 14, 30, 45, and 60 days after being exposed to the contaminated diets to evaluate brain and heart tissue samples for histopathological analysis.

### Behavioral tests

#### Learning behavioral test

After striped bass juveniles that received the HC-diet stopped their consumption (HC-fish) four organisms were marked and transferred to the aquarium with organisms that were exposed to the LC-diet. Also, 4 organisms exposed to the LC-diet were transferred to the aquarium with organisms being exposed to the HC-diet. The feeding scheme was maintained as the original setup of the experiment. The behavior of organisms was evaluated in both aquariums.

#### The dark/light box test

**Figure 5:**
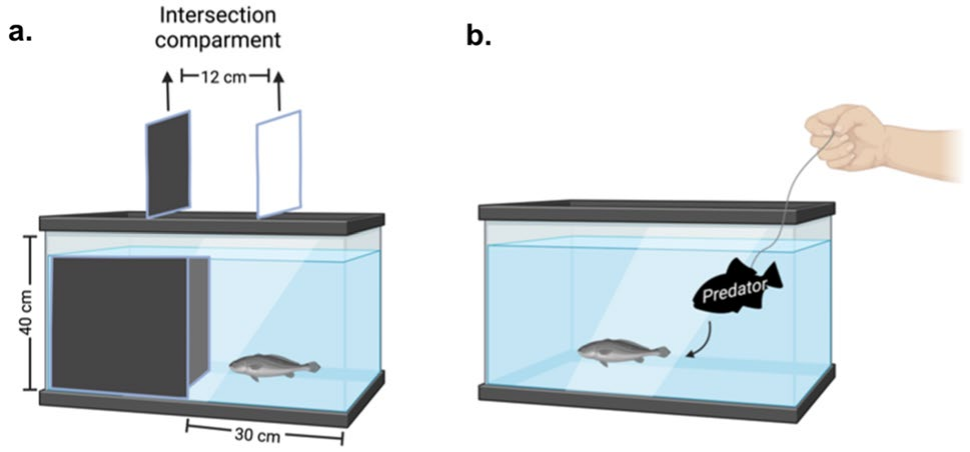
Standardized behavioral tests used to evaluate the effect of oral exposure of domoic acid on *Totoaba macdonaldi*. The dark/light trial aquarium (**a**) and the antipredator behavior test setup (**b**).

The experimental setup consisted of an aquarium divided into black and white sections. Fish were placed in an intersection compartment enclosed by two sliding doors located between both sides (Fig. 5a). After a 5 min habituation period, both doors were removed. The organisms were allowed to explore the tank freely, and its behavior was recorded for 15 min using a camera placed at the top of the aquarium. The test was applied to fish fed with the HC-diet (n = 7) and organisms fed with commercial pellets (n = 7). The behavior evaluation was conducted on days 0, 3, 7, 14, and 30 after the initiation of the exposure to the toxin. All the tests were performed at the same time from 13:00 and 15:00 h to avoid changes attributed to circadian activity patterns.

#### Antipredator behavior

The antipredator behavior was evaluated by the characterization of the escape response of DA-exposed and control organisms to a simulated predator attack. A silicon model of a lionfish (*Pterois sp*.) was used to simulate the predator attack. A new aquarium was implemented for the test. The “predator” was released generating a bow wave and the fish response was recorded with a camera placed at the top of the aquarium (Fig. 5b). The escape response was considered as the time between the entry of the predator to the aquarium and the flexion of the caudal peduncle of totoaba (C-startle reflex). The test was applied with the same organisms selected from the dark/light box test at days 0, 3, 7, 14, and 30 after the initiation of the exposure to the toxin. The behavioral footage was analyzed using CowLog (open-source software for coding behaviors from digital video).

### Euthanasia and dissection

Organisms were placed in cold water to reduce sensibility and a dose of 85 mg L^−1^ of eugenol was used as anesthesia. Organisms were dissected. The brain, heart, and liver were removed. A portion of each organ was fixed with 10% formol whereas another portion was frozen at − 80 °C for DA determination.

### HPLC–MS/MS domoic acid quantification

Samples for DA quantification were analyzed by liquid chromatography coupled to a triple quadrupole mass spectrometer (LC–MS/MS, Agilent 6470). Tissue samples stored at − 80 °C were thawed at room temperature. Samples were weighed in 2 mL Eppendorf tubes and the extraction solvent (MeOH 50%) was added in a 1:4 ratio. The samples were homogenized in a K-550-G vortex tube shaker for 2 min prior to centrifugation at 13,000 rpm for 8 min at 4 °C. Subsequently, the supernatant was recovered and passed through polypropylene 0.2 µm pore size filters. The filtrate was recovered in a 250 µL insert which was placed in 1.5 mL amber tubes where trifluoroacetic acid (TFA) was added to a final concentration of 0.15%. LC–MS/MS analysis was carried out according to the method of Mafra et al.^39^

### Histological analysis

The brain and heart samples of the MDA and diet bioassays were embedded in paraffin and sectioned at 3 µm using a manual microtome (Leica-RM2125 RT, IL USA). Transversal sections of the brain were obtained. Specifically, mesencephalon (optic tectum) and metencephalon (cerebellum) areas were analyzed. Sagittal cuts were performed on heart samples. The slides were stained with hematoxylin and eosin. The slides were observed using a Leica DM2000 microscope and images were obtained using the software Leica Acquire Software.

#### Serial sections

Seven organisms of *T. macdonaldi* that had been exposed to DA-contaminated feed for 45 days and seven organisms exposed to toxin-free feed were processed to perform serial cuts of the brains. The organisms were euthanized as described above and the whole brain was removed from the skull and fixed for a month in 10% formol. The brains were dehydrated, embedded in paraffin, and cut into 5 µm transverse sections. The sections were stained with hematoxylin and eosin.

Three DA-exposed brains and one control brain were chosen for photography documentation. Using computer edition, artifacts and other undesired structures, such as ventricular contents, were removed. Fifty slides of each analyzed brain were obtained, and five images of each section were obtained and were composed in one single figure using BioRender (https://biorender.com/). The structures that were lost when the brain was removed from the skull were represented with dotted lines in the images when necessary. The most representative figures of each brain were chosen according to the appearance of novel structures.

### Data analysis

A Wilcoxon Rank-Sum Test was used to compare the weight of the organisms from the diet bioassay at the beginning of the experiment and when the organisms rejected the diet (to evaluate the growth of organisms from the start of the bioassay to the time point where the organisms rejected the HC-diet (day 7 for striped bass and day 45 for totoaba). Growth parameters were evaluated using the following formulas:

$${\text{Weight}}\;{\text{gain}} = {\text{FW}} - {\text{IG}}$$

$${\text{Average}}\;{\text{daily}}\;{\text{weight}}\;{\text{gain}} = \left( {{\text{FW}} - {\text{IF}}} \right)/{\text{Days}}$$

$${\text{Specific}}\;{\text{growth}}\;{\text{rate}} = \left( {{\text{Ln}}\left( {{\text{FW}}} \right) = {\text{Ln}}\left( {{\text{IW}}} \right)} \right)*100$$

where: FW: the weight in grams at the end of the bioassay period, IW: the weight in grams at the beginning of the bioassay period, Ln: natural logarithm.

The change (in relative units; r.u.) of each behavioral biomarker (Bm) was assessed through the comparison of the response of each organism at the sampling time (tn) to the one measured at day 0 (t0) before the beginning of the exposition to DA ((Bm_tn_-B_to_)/Bm_t0_). For each behavioral biomarker, data of exposed and unexposed organisms were compared using paired Student’s t-test. Nonparametric Wilcoxon test was used when the normality and homoscedasticity assumptions were not fulfilled. A Wilcoxon Rank-Sum test was used for behavioral biomarkers of exposed organisms at different sampling points and was compared to the response at t = 0. Statistical significance was evaluated at α = 0.05 level. We used STATISTICA (Data Analysis Software System) Version 14 (www.statsoft.com) for the data analysis.

### Supplementary Information

Supplementary Information 1.

Supplementary Video 1.

Supplementary Video 2.

Supplementary Video 3.

## Data Availability

The data that supports the findings of this study are available from the corresponding author upon request.
