# Supplementary material for: Domoic acid affects brain morphology and causes behavioral alterations in two fish species
Source: Sci Rep. 2023 Dec 8;13:21729. doi: 10.1038/s41598-023-49041-0 (PMC10709449; doi:10.1038/s41598-023-49041-0)
Supplement: Supplementary file 1 — Supplementary Information 1. [file 41598_2023_49041_MOESM1_ESM.docx]

Domoic acid affects brain morphology and causes behavioral alterations in two fish species

Kassandra Beltrán-Solís^1^, Ernesto García-Mendoza^2*^, Samuel Sánchez-Serrano^3^ & Lus M. López^3^

^1^ Posgrado en Ecología Marina, Centro de Investigación Científica y de Educación Superior de Ensenada (CICESE).

^2^ Departamento de Oceanografía Biológica, Centro de Investigación Científica y de Educación Superior de Ensenada (CICESE).

^3^ Facultad de Ciencia Marinas, Universidad Autónoma de Baja California (UABC)

Correspondence and requests for material should be addressed to Ernesto García Mendoza (email: ergarcia@cicese.mx)

**
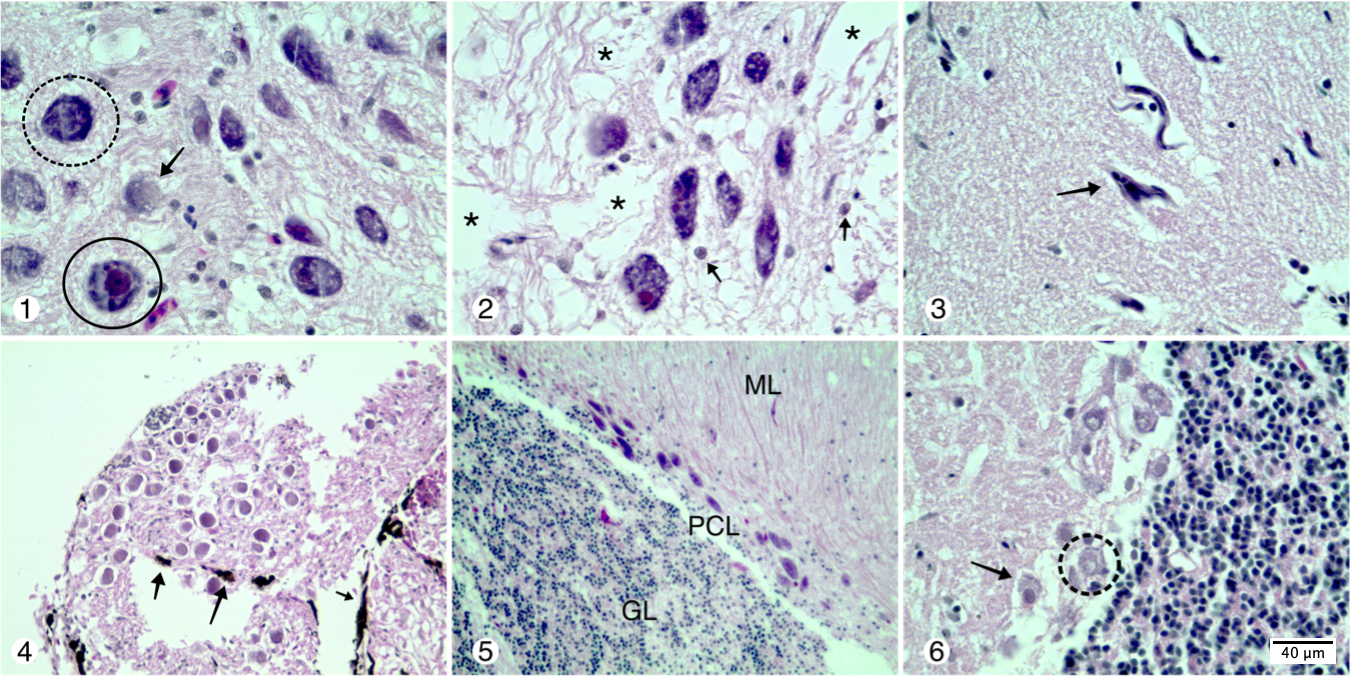
**

**Supplementary Figure 1.** Brain tissue of *Totoaba macdonaldi* after IP injection of 1.6 µgDA g^-1^. Optic tectum (OT; 1) Neuron with pyknotic nucleus and cytoplasm vacuolization (continuous circle). (2) Neuropil loss (asterisk) and inflammatory cells infiltration (arrows) in OT. (3) Neurons with fragmented nucleus and loss of membrane integrity in the OT. (4) Spongiform change of the neuropil and melanism (arrows) in the OT. (5) Cerebellum (CER; ML=molecular layer; PCL=Purkinje cell layer; GL=granular layer) separation between PCL and GL. (6) Purkinje cell with pyknotic nucleus (arrow) and cell with cytoplasm vacuolization (dotted circle) in CER.

**
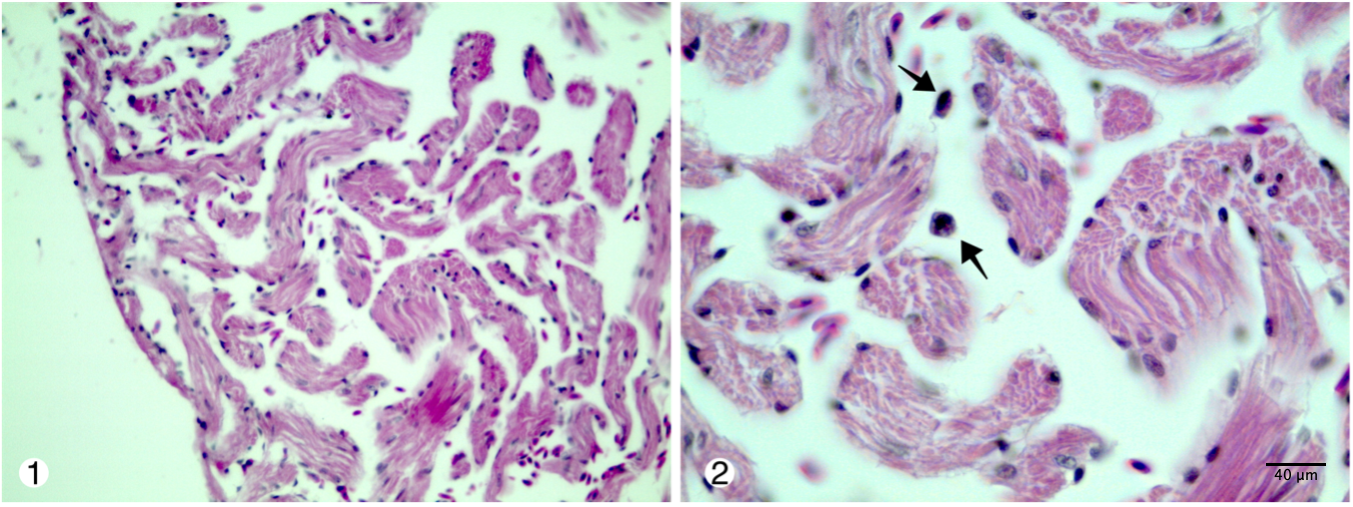
**

**Supplementary Figure 2.** Heart tissue of *Totoaba macdonaldi* after IP injection of ≥ 1.6 µgDA g^-1^. (1) Myocardium with separation between muscle fibers. (2) Inflammatory cells infiltration (arrows).


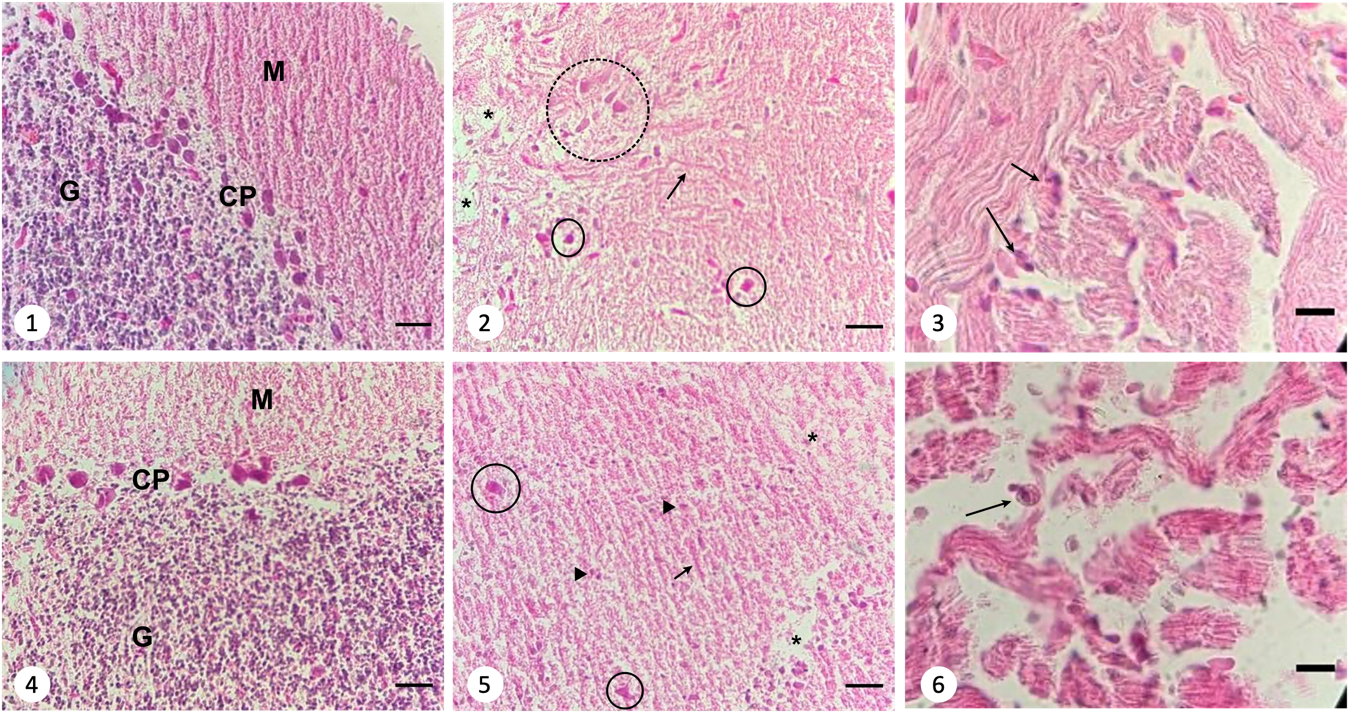


**Supplementary Figure 3.** Brain and heart tissue of *Totoaba macdonaldi* after micropipette-guided drug administration (MDA) of 1.6 µgDA g^-1^ (Panels 1-3) 24 hours after gavage. (Panels 4-6) 72 hours after gavage. (1) Cerebellum (CER) without tissue alterations. M=molecular layer. CP=Purkinje cell layer. G=granular layer. (2) Optic tectum (OT). Neurons with condensed nucleus (pyknosis, continuous circle). Neurons with pale cytoplasm and irregular borders (dotted circle). Neuropil loss (asterisk). (3) Myocardium. Necrotic cardiomyocytes (arrows). (4) CER at 72 hours post gavage without morphological alterations. (5) Pyknotic neurons (circles), glial cells (arrowheads), and neuropil loss (asterisk) in the OT (6) Separation between muscle fibers. Inflammatory cell infiltration (arrow) in the myocardium. Scale bar = 40 μm.


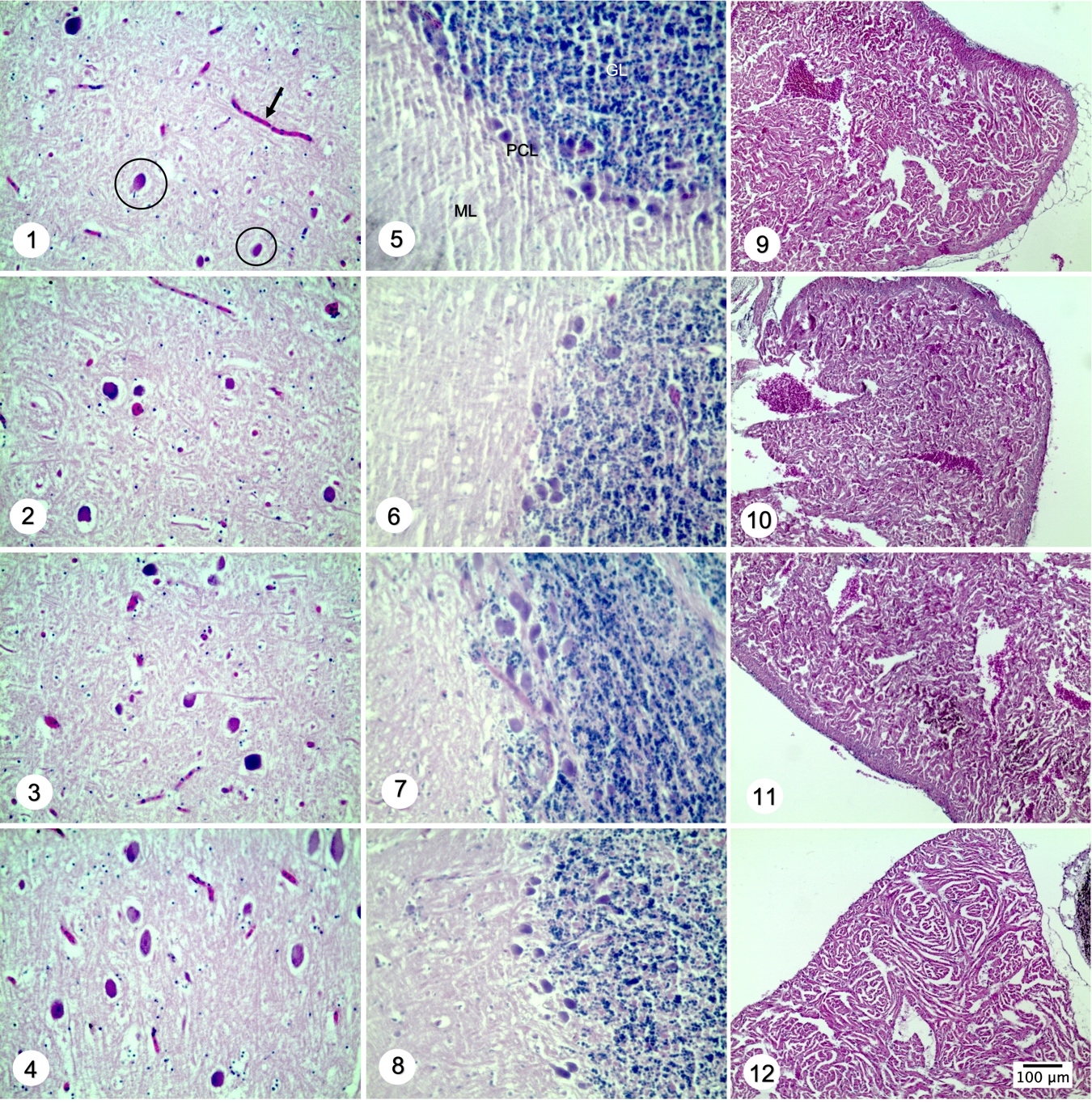


**Supplementary Figure 4.** Brain and heart tissue of *Totoaba macdonaldi* after feeding trial with LC-diet. (Panels 1-4) Neurons in the optic tectum (OT) with normal morphology. (1) OT day 0. (2) OT day 7. (3) OT day 14. (4) OT day 60. (Panels 5-8) Cerebellum (CER) without tissue damage. ML=molecular layer. PCL=Purkinje cell layer. GL=granular layer. (5) CER day 0. (6) CER day 7. (7) CER day 14. (8) CER day 60. (Panels 9-12) Heart tissue (HT) with normal morphology. (9) HT day 0. (10) HT day 7). (11) HT day 14. (12) HT day 60.


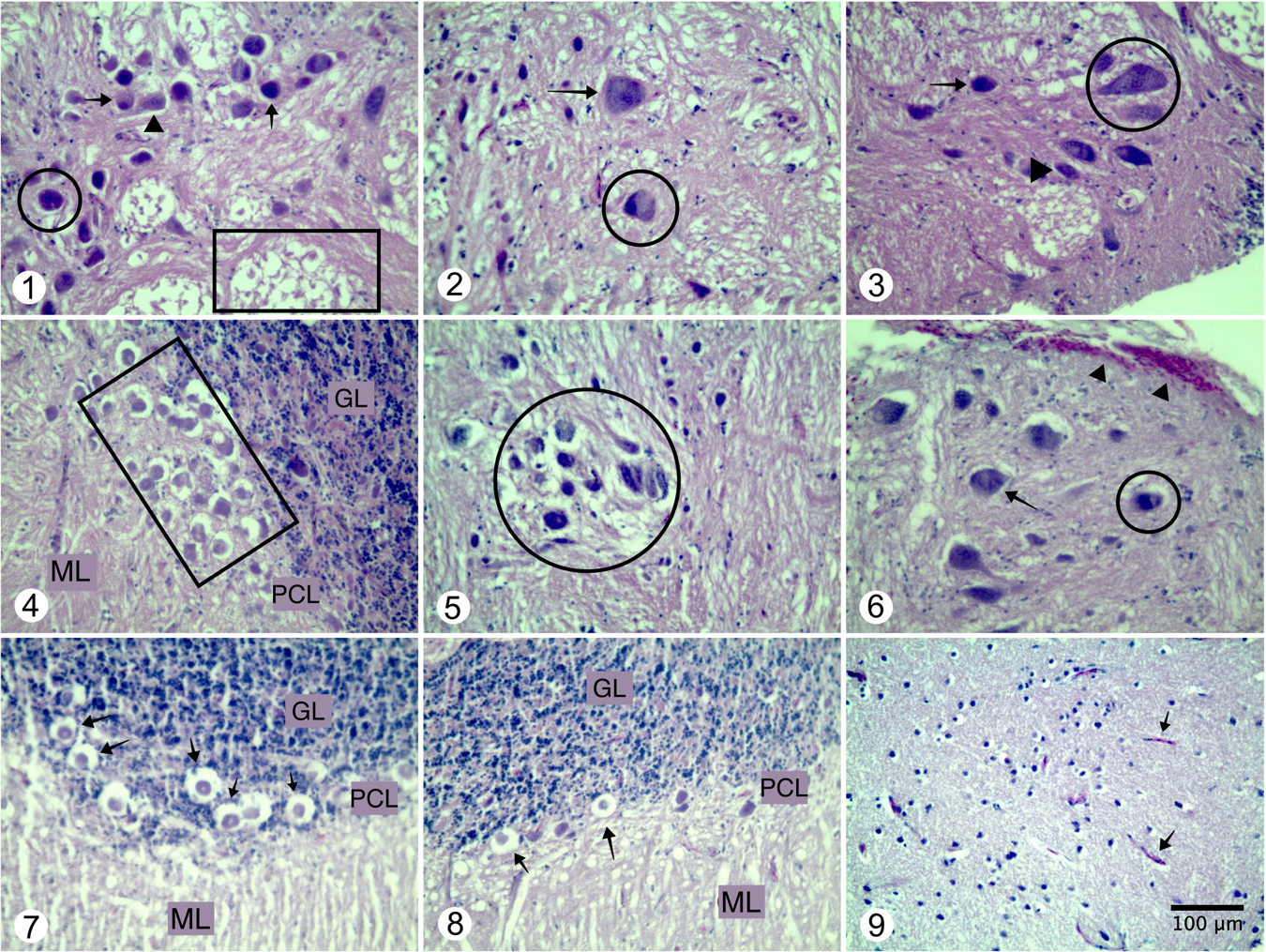


**Supplementary Figure 5.** Brain tissue of *Morone saxatilis* after feeding trial with HC-diet (Panels 1-2) Brain tissue at 7 days after consuming DA-contaminated food. (1) Optic tectum (OT). Neurons with condensed nucleus (pyknosis) (arrows). Neuron with fragmented nucleus (karyorrhexis) (circle). Liquefactive necrosis characterized by spongiform change of neuropil (rectangle). (2) Neuron with condensed nucleus (circle). Neuron with fragmented nucleus (arrow) in the OT. (Panels 3-4) Brain tissue at 14 days after consuming DA-contaminated food. (3) OT with neurons at different stages of oncotic necrosis: pyknosis (arrow), karyorrhexis (arrowhead), and karyolysis (circle). (4) Cerebellum (CER). ML=molecular layer. PCL=Purkinje cell layer. GL=granular layer. Rectangle: Purkinje cells vacuolization. (Fig. 5-7) Brain tissue at 50 days after consuming DA-contaminated food. (5) OT with liquefactive necrosis (circle). (6) Necrotic neurons with pyknosis (circle) and karyorrhexis (arrow) in the OT. (7) CER with vacuolization at Purkinje cells layer(arrows). (Panels 8-9) Brain tissue at 60 days after consuming DA-contaminated food. (8) CER with vacuolization at Purkinje cells layer (arrow). (9) OT with gliosis at astrocytes and hyperemic capillaries (arrows).


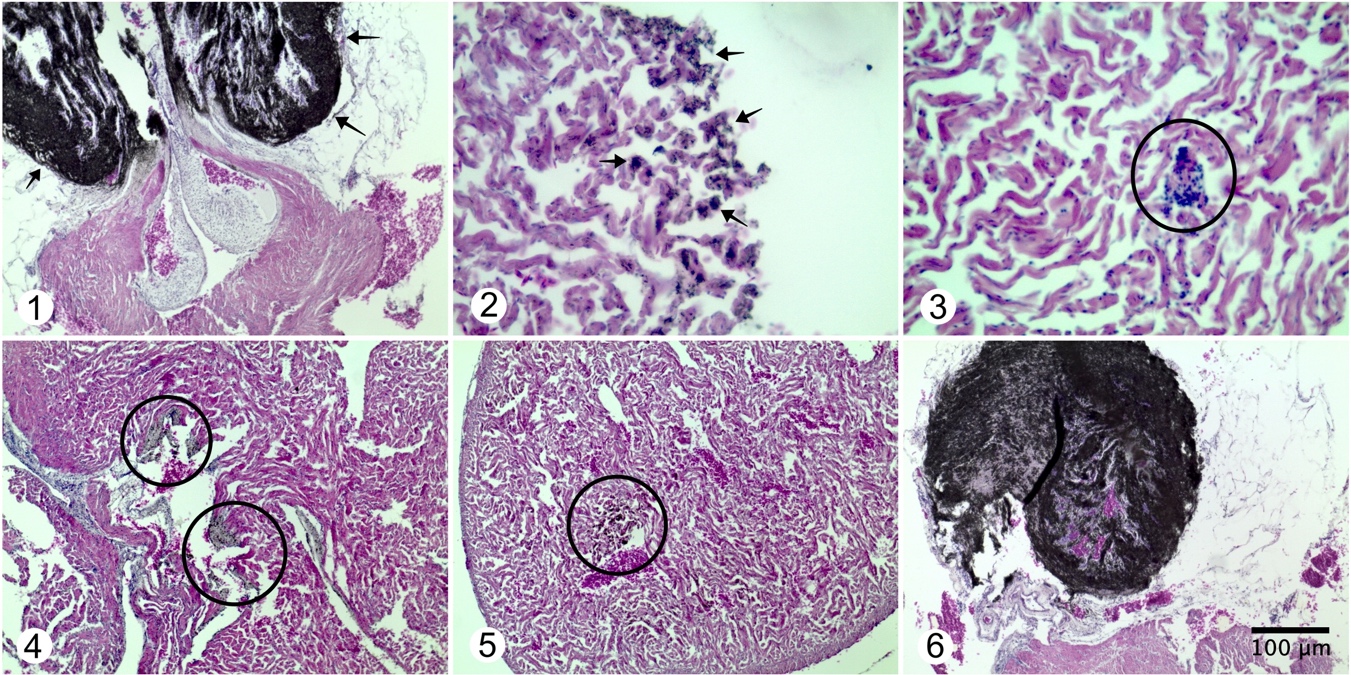


**Supplementary Figure 6.** Heart tissue of *Morone saxatilis* after feeding trial with the HC-diet (Panels 1-2) Heart tissue at 7 days after consuming DA-contaminated food. 1) Atrial melanism (arrows). 2) Necrotic cardiomyocytes (arrows). 3) Heart tissue at 14 days after consuming DA-contaminated food. Nuclei accumulation in the myocardium (circle). 4) Cardiac muscle at 28 days after consuming DA-contaminated food. Melanism in muscle fibers (circles). (Panels 5-6) Heart tissue at 40 days after consuming DA-contaminated food. 5) Myocardium. Necrotic cardiomyocytes. 6) Atrial melanism.


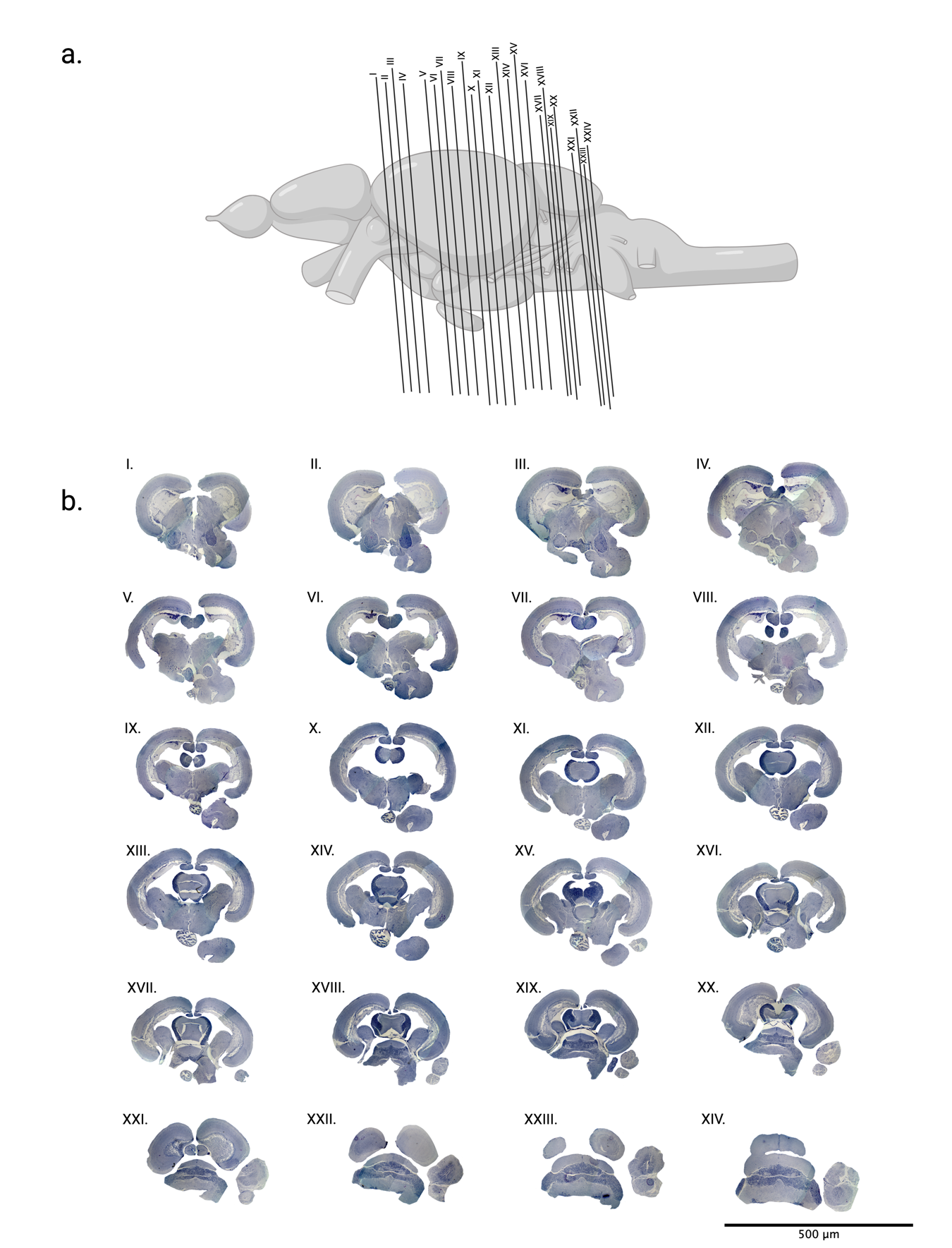


**Supplementary Figure 7.** Brain serial cross-sections of a non-exposed to DA *Totoaba macdonaldi* juvenil (a) The lateral view of the teleost brain indicates the positions of cross sections presented in panel (b) The cross-series sections were arbitrarily labeled from the cephalic region to the caudal region of the brain.


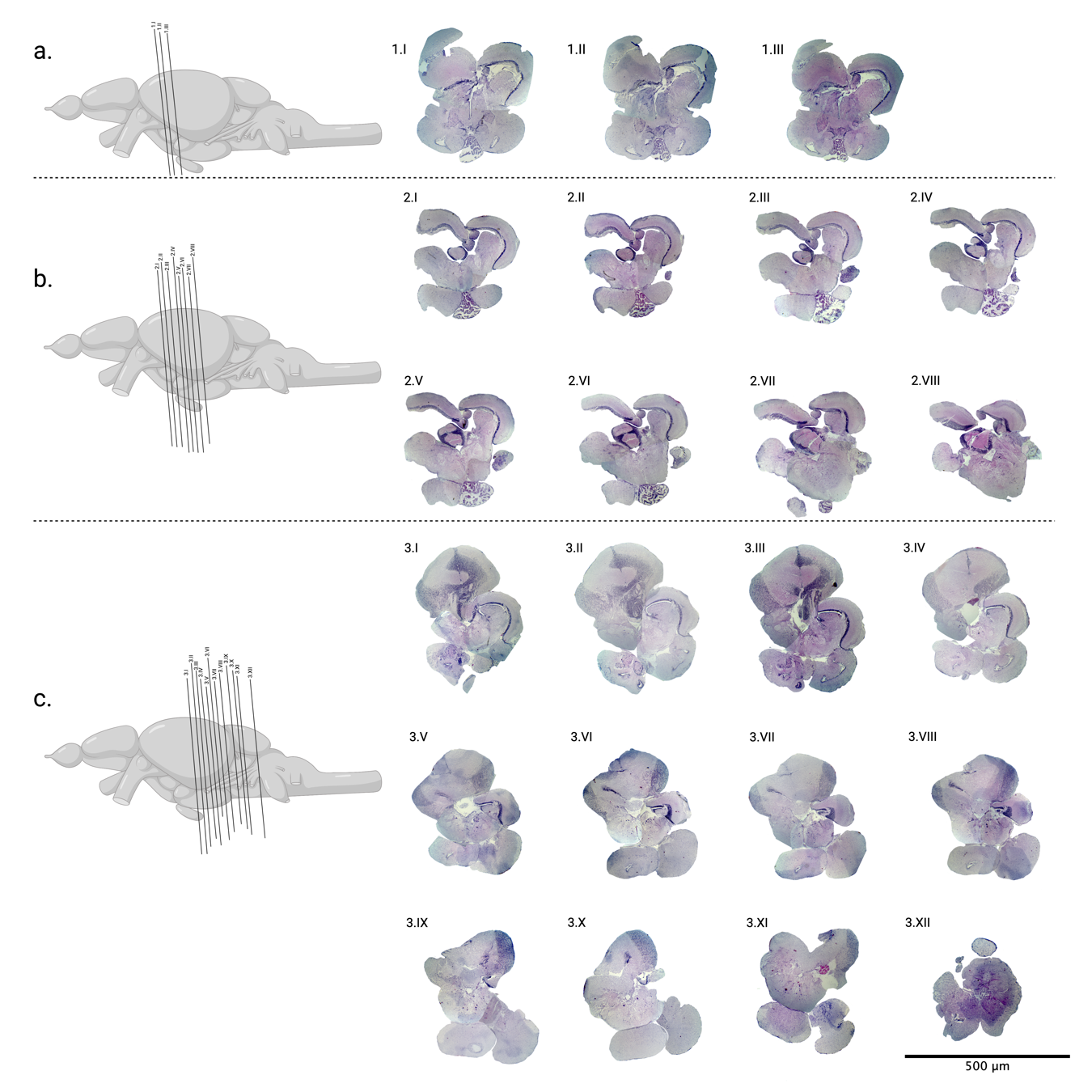


**Supplementary Figure 8.** Brain serial cross-sections of totoaba juveniles exposed to DA-contaminated feed. The lateral view of the teleost brain indicates the position of levels illustrated in the following series of cross-sections. Each panel (a, b, c) represents an individual organism to illustrate the different kinds of malformations on the right hemisphere induced by DA consumption.


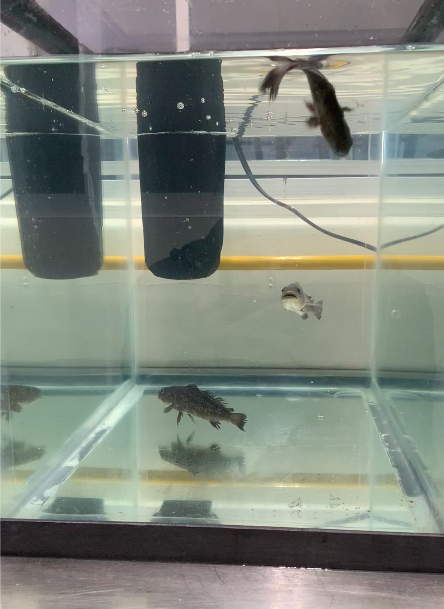


**Video 01.** Neurotoxic signs on *Totoaba macdonaldi* after IP injection of 3.2 µgDA g^-1^. The organisms presented disorientation, swam in circles, spirals, or upside down.


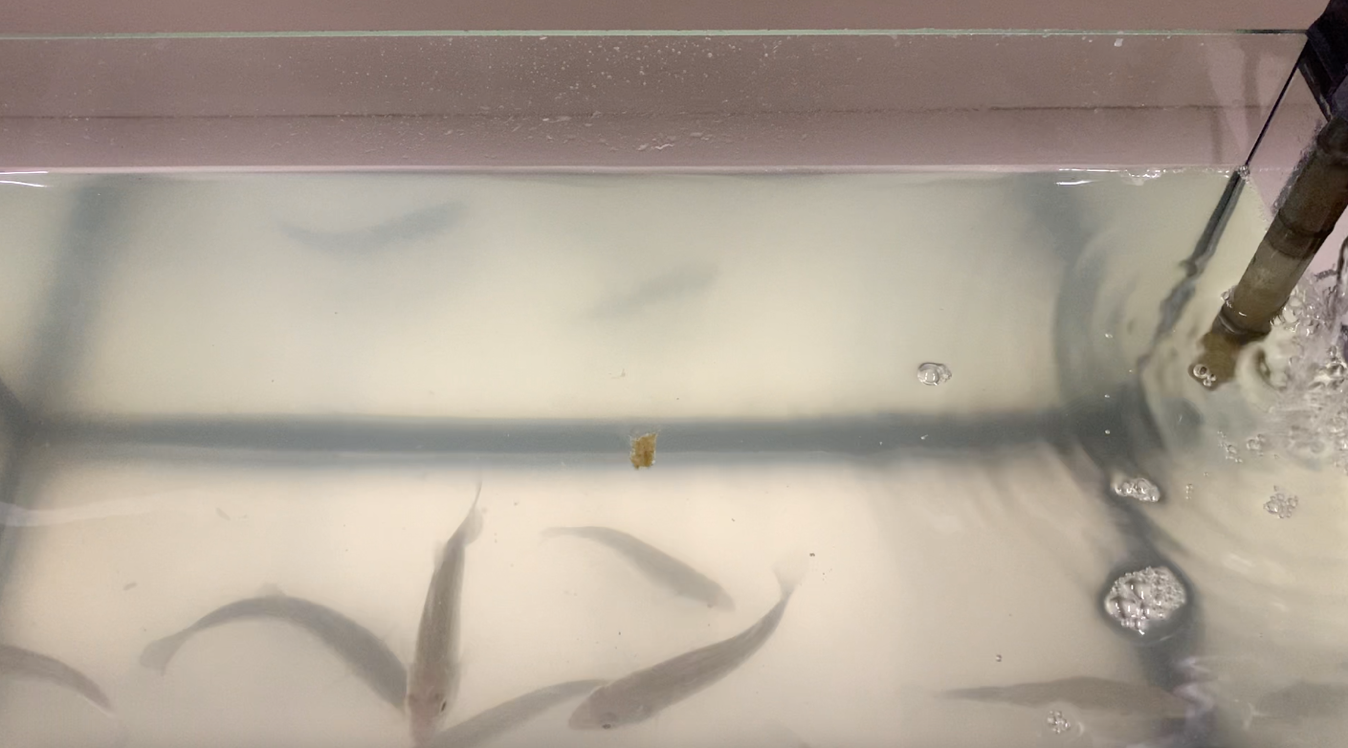


**Video 02.** *Morone saxitilis* rejecting the HC-diet after 7 days of consumption.


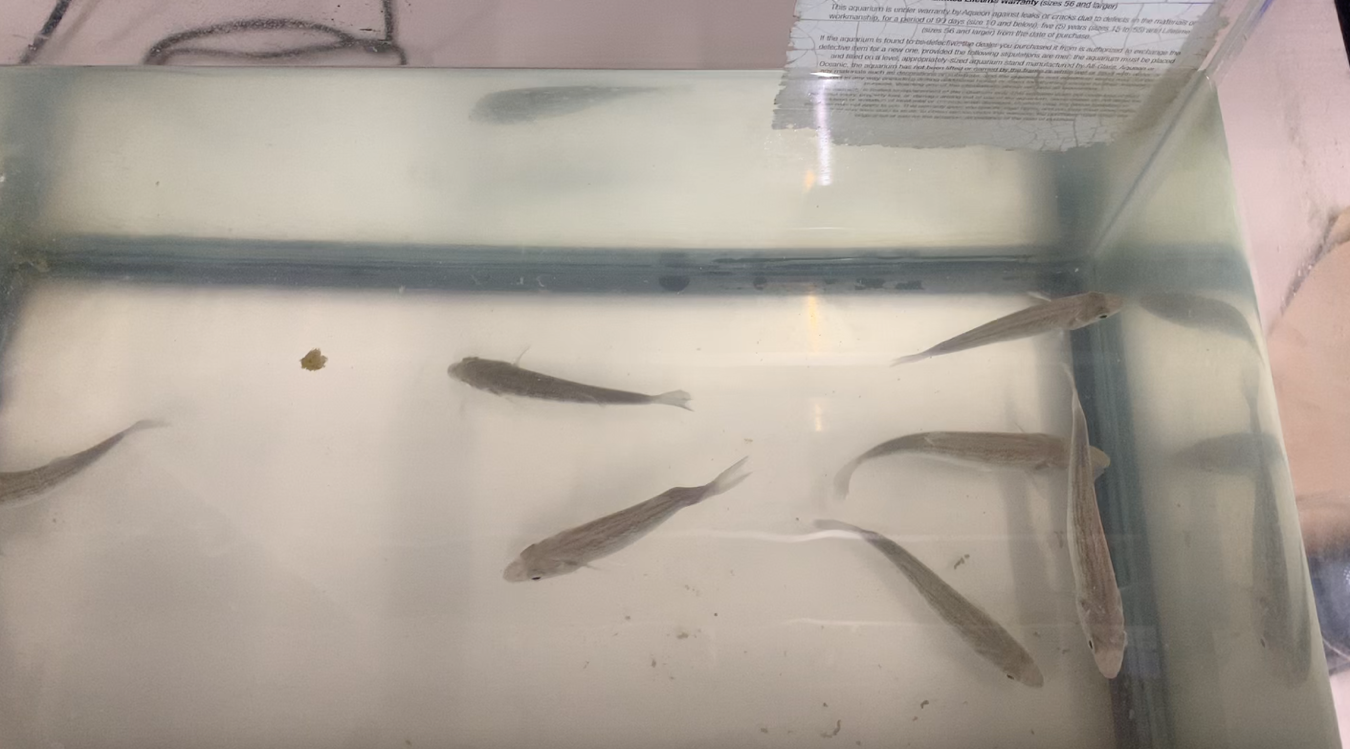


**Video 03.** Learning behavioral test on *Morone saxatilis* organisms. HC-fish rejecting the HC-diet (first approaching fish). LC-fish consuming the HC-diet (second approaching fish).
